# Differential Actions of Muscarinic Receptor Subtypes in Gastric, Pancreatic, and Colon Cancer

**DOI:** 10.3390/ijms222313153

**Published:** 2021-12-05

**Authors:** Alyssa Schledwitz, Margaret H. Sundel, Madeline Alizadeh, Shien Hu, Guofeng Xie, Jean-Pierre Raufman

**Affiliations:** 1 Department of Medicine, Division of Gastroenterology & Hepatology, University of Maryland School of Medicine, Baltimore, MD 21201, USA; alyssa.schledwitz@som.umaryland.edu (A.S.); madeline.alizadeh@som.umaryland.edu (M.A.); shu1@som.umaryland.edu (S.H.); gxie@som.umaryland.edu (G.X.); 2 Department of Surgery, University of Maryland School of Medicine, Baltimore, MD 21201, USA; maggie.sundel@som.umaryland.edu; 3 The Institute for Genome Sciences, University of Maryland School of Medicine, Baltimore, MD 21201, USA; 4 VA Maryland Healthcare System, Baltimore, MD 21201, USA; 5 Marlene and Stewart Greenebaum Comprehensive Cancer Center, University of Maryland School of Medicine, Baltimore, MD 21201, USA; 6 Department of Biochemistry and Molecular Biology, University of Maryland School of Medicine, Baltimore, MD 21201, USA

**Keywords:** muscarinic receptors, gastric cancer, pancreatic cancer, colorectal cancer, acetylcholine

## Abstract

Cancers arising from gastrointestinal epithelial cells are common, aggressive, and difficult to treat. Progress in this area resulted from recognizing that the biological behavior of these cancers is highly dependent on bioactive molecules released by neurocrine, paracrine, and autocrine mechanisms within the tumor microenvironment. For many decades after its discovery as a neurotransmitter, acetylcholine was thought to be synthesized and released uniquely from neurons and considered the sole physiological ligand for muscarinic receptor subtypes, which were believed to have similar or redundant actions. In the intervening years, we learned this former dogma is not tenable. (1) Acetylcholine is not produced and released only by neurons. The cellular machinery required to synthesize and release acetylcholine is present in immune, cancer, and other cells, as well as in lower organisms (e.g., bacteria) that inhabit the gut. (2) Acetylcholine is not the sole physiological activator of muscarinic receptors. For example, selected bile acids can modulate muscarinic receptor function. (3) Muscarinic receptor subtypes anticipated to have overlapping functions based on similar G protein coupling and downstream signaling may have unexpectedly diverse actions. Here, we review the relevant research findings supporting these conclusions and discuss how the complexity of muscarinic receptor biology impacts health and disease, focusing on their role in the initiation and progression of gastric, pancreatic, and colon cancers.

## 1. Introduction

**Figure 1:**
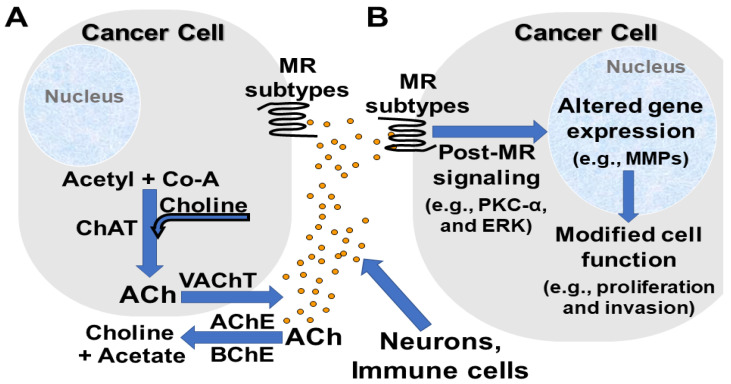
Capability of neurons, immunocytes, and cancer cells to produce and release acetylcholine (ACh) that promotes cancer progression. (**A**). Neurons, immunocytes, and cancer cells express enzymes (e.g., choline acetyltransferase, ChAT) and transporters (e.g., vesicular acetylcholine transporter, VAChT) necessary to produce and release ACh. Acetyl- (AChE) and butyrylcholinesterases (BChE) in the extracellular space rapidly hydrolyze ACh to acetate and choline. (**B**). ACh activates muscarinic receptor (MR) subtypes expressed by adjacent cancer cells. Post-muscarinic receptor signaling activates several protein kinases (e.g., protein kinase C-α, PKC-α), and transcription factors (e.g., extracellular signal-regulated protein kinase 1/2, ERK1/2), thereby altering the expression of genes that encode for proteins that modify cell function and promote cancer cell proliferation, survival, migration, invasion, and metastasis (e.g., matrix metalloproteinases [MMPs] like MMP1, MMP7, and MMP10).

The role of neurotransmitters in modulating cancer cell behavior has emerged as a major focus of investigation and a promising avenue for developing novel therapeutics [1,2,3]. This takes on particular importance for cancers of the organs in the gastrointestinal (GI) tract that are innervated by both the central and enteric nervous systems [1,4]. The complexity of this field is highlighted by evidence that so-called neurotransmitters (e.g., acetylcholine, ACh) can be produced and released by non-neural cells (e.g., immunocytes, stromal cells, and cancer cells themselves) (Figure 1). The actions of these agents are further modulated by the differential distribution and regulation of their receptors and the enzymes required for their hydrolysis (e.g., acetyl- and butyrylcholinesterases) [5] (Figure 1). Understanding the dynamics of these interactions and their underlying mechanisms is likely to open the door to developing new biomarkers and therapeutic modalities.

Here, because there is an abundance of novel information and the incidence of tumors in these organs is increasing in younger populations [6,7], we focus on the differential actions of activating muscarinic receptor subtypes on the evolution and progression of cancers of the stomach, pancreas, and colon. Innovative therapies for these cancers are urgently needed since effective treatment options are limited once adenocarcinomas arising from these organs progress to surgically unresectable, advanced, and invasive stages [8,9]. Notably, for these GI cancers, even novel immunomodulators, like PD-1 checkpoint inhibitors, appear to hold little promise for successful treatment as their effects tend to be modest and lack durability [10,11]. Thus, both the gaps in current knowledge and the need for new therapeutic approaches are highlighted in this review.

To provide a comprehensive overview of the role muscarinic receptor subtypes and their ligands play in the progression of these GI cancers, we divided this review into discrete sections. Focusing on gastric, pancreatic, and colon cancer, we review information regarding the distribution of neural and non-neural sources of ACh and other agents reported to modulate muscarinic receptor function by direct or allosteric interactions, the distribution and expression of the five muscarinic receptor subtypes (M_1_R–M_5_R, encoded by *CHRM1*–*CHRM5*), and their differential roles in modulating cancer cell behavior. Nonetheless, it may be difficult to compartmentalize these highly integrated actions. To unravel their complexity, a systems biology approach may be helpful and, based on the implications for cancer therapeutics, appears warranted.

## 2. Muscarinic Receptor Ligands

### 2.1. Overview

Whereas ACh was commonly thought to be synthesized and released from neurons only, emerging evidence from several laboratories indicates that the cellular machinery required to synthesize and release ACh is also present in immune, cancer, and other cell types (Table 1), as well as in lower organisms (e.g., bacteria) that comprise the gut microbiome. In this section, we review the challenges faced by investigators attempting to measure tissue concentrations of ACh and their changes over time and the evidence that, by allosteric and other mechanisms, cholesterol, and its metabolic by-products (e.g., bile acids), interact with and alter the function of G protein-coupled receptors, including muscarinic receptors.

### 2.2. Neuronal and Non-Neuronal Acetylcholine

Although initially considered solely a neurotransmitter, abundant evidence obtained over the past 20 years indicates that many non-neuronal cells possess the capacity to synthesize and release ACh [12,13,14,15,16,17,18] (Table 1). This is suggested by identifying acetylcholine, as well as its derivatives in tissues; typically, these molecules are identified using either radiolabeled choline, a precursor to ACh, or an electrochemical detection method such as high-performance liquid chromatography with electrochemical detection (HPLC-ED) [12]. Radiolabeled choline was detected in keratinocytes [19] and, using similar methods, choline and glycerophosphocholine were detected in human [20] and chicken cardiomyocytes [21]. In fact, it is conceivable that non-neuronal ACh plays a larger and more important role in modulating the behavior and aggressiveness of GI cancer cells than does ACh derived from classical neuronal release. For example, increased radiolabeled choline uptake in human urothelial cancers [22] suggests neoplastic epithelial cells may have an augmented need for choline to produce ACh. Likewise, investigators used HPLC-ED to detect ACh production and release from H508 and Caco-2 human colon cancer cell lines [12].

Nonetheless, relevant evidence for non-neuronal ACh production and/or release must be examined thoughtfully and critically. By itself, the presence of an enzyme important for ACh synthesis, e.g., choline acetyltransferase (ChAT), does not provide sufficient evidence that ACh is produced and released at concentrations that will effectively alter the function of neighboring cells by autocrine or paracrine mechanisms (Figure 1). Tissue diffusion rapidly dissipates released ACh, thereby making it difficult to measure tissue concentrations of ACh reliably. Unlike other neurotransmitters, no technique can fix ACh to tissue to facilitate immunohistochemical quantification [18]. Moreover, enzymes that efficiently hydrolyze ACh, e.g., acetylcholinesterase (AChE), limit the duration that bioactive concentrations are attained and maintained. Hence, it is important to measure tissue levels of ACh over time-this remains technically challenging [12].

### 2.3. Bile Acids as Physiological Bioactive Muscarinic Receptor Ligands

Bile acids comprise a large family of cholesterol derivatives that can be further modified in the gut by bacterial hydroxylases and in the liver by conjugation, primarily with glycine and taurine, and sulfation. The major effect of these structural modifications is to alter the amphipathic nature of these agents, which for decades were considered only to function in the digestion and absorption of lipids. Over the past 22 years, evidence has emerged from several laboratories indicating that selected bile acids are, in fact, signaling molecules that, amongst other actions, interact with muscarinic receptors and modulate post-receptor signaling [37,38,39]. Although the molecular determinants that mediate bile acid interactions with muscarinic receptors remain obscure, modeling has suggested the possibility that molecular mimicry between bile acid and ACh structures may explain competition for muscarinic receptor binding sites [40]. It is also conceivable, if not more likely, that bile acids exert their effects by allosteric modulation of ligand-muscarinic receptor interactions. Not surprisingly, cholesterol, the parent molecule for bile acid synthesis, shares structural similarity with bile acids. Notably, cholesterol was shown to modulate the behavior of Class A G protein-coupled receptors, including M_1_R, allosterically [41].

**Figure 2:**
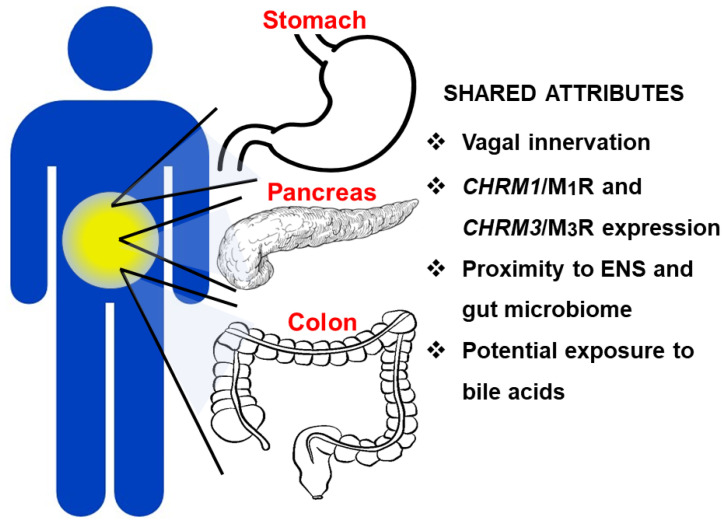
Key attributes shared by the stomach, pancreas, and colon that facilitate and promote cancer progression and metastasis. These anatomically proximate GI organs share vagal innervation, expression of *CHRM1*/M_1_ and *CHRM3*/M_3_ subtype muscarinic receptors, and exposure to luminal bile acids and the bacteria, viruses, and fungi that comprise the gut microbiome; these shared attributes can promote the development and progression of adenocarcinomas. ENS, enteric nervous system.

Bile acids, released from the liver and gallbladder into the common bile duct, can access gastric, pancreatic, and colonic adenocarcinomas via the GI lumen, respectively, by refluxing into the stomach and pancreatic duct, and spilling into the colon (Figure 2) [42,43]. Under certain circumstances, including prior cholecystectomy resulting in loss of bile storage in the gallbladder, intestinal surgery rerouting bile flow, and deficient intestinal release of fibroblast growth factor-19 (FGF19) resulting in unrestrained bile acid production by the liver, sustained elevation of bile acid levels in the pancreatic duct, stomach, and colon have the potential to alter the function of both normal and neoplastic epithelial cells by muscarinic mechanisms [44].

In several studies employing colon cancer cell lines in vitro, deoxycholic and lithocholic acids, and their glycine and taurine conjugates, modulate muscarinic receptor activation [37,40,44]. This line of investigation was initiated by the surprising observation that a bile acid, lithocholyltaurine, competed with a cholinergic agonist, carbamylcholine, for binding to the same muscarinic receptors on gastric mucosal cells [45]. Subsequently, the functional interaction of bile acids with muscarinic receptors was confirmed independently by investigators studying bile acid-induced cardiac arrhythmias [46]. Although the biological implications of the functional interaction of bile acids with muscarinic receptors are broad [44], perhaps the most important clinical impact is on gastrointestinal neoplasia. In human H508 colon cancer cells that express high levels of M_3_R, lithocholyltaurine at concentrations achievable in the human colon [47] caused a dose-dependent increase in cell proliferation [37], effects not observed in human SNU-C4 colon cancer cells that lack M_3_R expression [37]. Using stool obtained from the cecum of deceased humans, Hamilton et al., showed that bile acids in the proximal colon achieve concentrations in the range of 10–100 µM [47]. Bile acid concentrations are likely higher in living persons and in those following cholecystectomy or with ileal disease or resection that disrupts the enterohepatic circulation of bile acids. Notably, these ‘physiological’ concentrations of fecal bile acids were shown to stimulate colon cancer cell proliferation in vitro [47].

ACh and taurine and glycine conjugates of lithocholic and deoxycholic acids induce cell proliferation by post-receptor signaling involving transactivation of epidermal growth factor receptors (EGFR) and post-EGFR p44/42 mitogen activated protein kinase (MAPK) signaling [38]. EGFR transactivation is mediated by matrix metalloproteinase-7 (MMP7)-catalyzed release of heparin binding-EGF-like growth factor (HB-EGF), an EGFR ligand [48]. Post-M_3_R signaling also involves the activation of protein kinase C-α and p38 MAPK, with evidence that potentiating crosstalk between post-receptor signaling pathways augments cell proliferation, migration, and invasion [49].

## 3. Muscarinic Receptor Subtypes, Signaling, and Anatomic and Cellular Distribution

### 3.1. Overview

Since muscarinic receptors represent an ancient form of signaling, it is not surprising that the genetic lineage for the ACh receptors family in general, and muscarinic receptors (mAChR) in particular, can be traced to the earliest life forms with >80% receptor homology preserved amongst vertebrates [50]. Although muscarinic receptor subtypes are expressed in all vertebrates examined, there are important differences. For example, M_1_R is not expressed in teleosts or chickens and other birds [50]. This finding suggests that the actions regulated by M_1_R are not critical for the life and health of these organisms, that redundant actions among the muscarinic receptors compensate for the absence of M_1_R, or that M_1_R functions are assumed by other signaling pathways.

In the following sections, we consider the tissue distribution and actions of each of the five muscarinic receptor subtypes (Table 2). By way of overview, it is important that this family of receptors is segregated into two subfamilies, M_1_R, M_3_R, and M_5_R (sometimes referred to as MR_odd_), and M_2_R and M_4_R (MR_even_). The former, coupled to G_q/11_, signal initially via changes in cellular phospholipids and calcium. The latter, coupled to G_i/o_, signal via changes in cellular cAMP. All five muscarinic receptor subtypes are expressed in the brain, where M_1_R, M_2_R, and M_4_R predominate. M_3_R appears to play a larger role outside the brain, including throughout the GI tract where it appears to play a major role in regulating normal physiological processes, like fluid and electrolyte secretion [50].

### 3.2. CHRM1/M_1_R

*CHRM1*/M_1_R was the first muscarinic receptor subtype successfully knocked out globally in mice. Consequently, the impacts of both global M_1_R deficiency and, more recently, conditional M_1_R deficiency in a number of organs, have permitted elucidation of M_1_R actions in considerable detail [67]. M_1_R is the dominant muscarinic receptor subtype in the central nervous system; it is also expressed in the respiratory epithelium and skin, and widely in the GI tract where it contributes to the regulation of salivary, gastric, and pancreatic secretion.

Located largely in forebrain areas such as the neocortex, hippocampus, and striatum, M_1_Rs are the primary muscarinic receptors implicated in higher-level cognitive functions, including learning and memory [68,69]. M_1_R inactivation results in hyperactive mice with elevated dopamine levels, which potentially explains impaired cognitive processing. These findings underly the utility of M_1_Rs as potential therapeutic targets for Parkinson’s disease and psychiatric disorders (e.g., schizophrenia) [70].

Although not the functionally dominant muscarinic receptor subtype in the heart, M_1_R plays a role in catecholamine-mediated cardiac activity and may be upregulated in certain conditions, e.g., chronic atrial fibrillation [71,72]. M_1_R, expressed by the normal prostate [73,74], is upregulated in animal and in vitro models of prostate cancer [75,76]. M_1_R expression by salivary and pancreatic tissue is important for the regulation of fluid and electrolyte secretion from sublingual and submandibular glands [77,78] and amylase secretion from pancreatic acini [79], respectively. As discussed in greater detail below, the relationship between M_1_Rs and GI cancers appears protective; M_1_Rs act as tumor suppressors for pancreatic ductal and colon adenocarcinoma [51,80].

### 3.3. CHRM2/M_2_R

*CHRM2*/M_2_R is prominently expressed in the brain, heart, skin, bladder, smooth muscle, and GI tract [34]. In the brain, the highest levels of M_2_R expression are in the cerebral cortex, forebrain, caudate and putamen, and thalamus, although M_2_R is also expressed in the brainstem and cervical spinal cord [55]. M_2_R is the predominant muscarinic receptor subtype expressed in the heart, playing key regulatory roles in cardiovascular electrophysiology, e.g., modulating heart rate and contractility by altering the activity of inwardly rectifying potassium channels and inducing vascular dilation via nitric oxide release, respectively [65]. In the lungs, M_2_R plays a role in airway responsiveness; M_2_R dysregulation augments irritant- and inflammation-induced bronchoconstriction [81,82].

Within the GI tract, M_2_R is the primary muscarinic receptor expressed by smooth muscle cells and interstitial cells of Cajal in the enteric nervous system and modulates GI motility [34,54]. Additionally, knockout mouse models were used to show that M_2_R and M_4_R expression plays a role in regulating the autoinhibition of ileal ACh release [56]. Although potential roles for M_2_R expression and activation are reported for brain (glioblastoma) and small cell lung cancers [34], to date, M_2_R has not been reported to play a role in modulating the behavior of any GI tract cancer.

### 3.4. CHRM3/M_3_R

The M_3_ receptor has a wide anatomic distribution, notably in the GI tract, bladder, eye, central and peripheral vasculature, and exocrine and endocrine glands. Though not the proportionally dominant muscarinic receptor within the GI tract, M_3_Rs are functionally important, inducing smooth muscle contraction and affecting motility in the stomach, ileum, and colon [83,84]. M_3_R similarly regulates smooth muscle contraction within the bladder, lung, and eye and, in these locations, is likely the primary mediator of muscarinic receptor-induced contraction [85,86,87,88]. In the vasculature, M_3_Rs are localized within the endothelium and smooth muscle and regulate endothelial-dependent relaxation and dilation of blood vessels, mediated by nitric oxide [89,90].

M_3_R has secretory functions throughout the body. In concert with M_1_R and M_5_R, respectively, M_3_R contributes to pepsinogen and gastric acid secretion [52,53]. M_3_R plays an important role in endocrine and exocrine pancreatic secretion, with many potential clinical applications [79,91]. Hyperactivation of M_3_Rs induces pancreatitis in mice, and allosteric modulation of M_3_R activity can normalize glucose homeostasis in obese mice [92,93]. The contribution of M_3_Rs to exocrine gland secretion, and, in particular, to salivary gland secretion, helps explain the common side effect of dry mouth (xerostomia) associated with the use of muscarinic receptor antagonists [94] and the finding of anti-M_3_R antibodies in Sjogren’s syndrome [95,96]. As discussed below, regarding its oncologic role, M_3_R activation enhances gastric, pancreatic, and colon cancer cell proliferation, and is a biomarker for metastases and a poor prognostic for these cancers [60,61,97].

### 3.5. CHRM4/M_4_R

Like M_2_R, M_4_R is a G_i_ protein-coupled receptor that reduces cellular levels of cAMP. M_4_R is expressed primarily in the stomach, duodenum, small intestine, and brain, especially in the striatum, however relatively high expression of *CHRM4* mRNA is also reported in the spleen [63,65,98]. M_4_R is strongly expressed in the stomach, where it was recently discovered to play a role in gastric acid secretion, a process primarily driven by M_3_R activation. Although the underlying mechanism and its relevance to human biology remains unclear, it is hypothesized that M_4_R activation inhibits the release of somatostatin, an inhibitory bioactive peptide, from gastric and duodenal D cells, thereby disinhibiting M_3_R-mediated gastric acid secretion from parietal cells [57].

In recent years, there has been more interest regarding the role of M_4_R in the brain. M_4_R activation negatively regulates dopamine release from the striatum via signaling dependent on expression of CB_2_ cannabinoid receptors; induced depression of dopamine release is sustained ex vivo after removal of M_4_R agonists and is accompanied in vivo by anti-psychotic-like behavior in mice [64,98]. Imbalances in cholinergic transmission contribute to the development of many neuropsychiatric disorders including Alzheimer’s disease and schizophrenia, therefore, M_4_R is under active investigation as a potential drug target. Notably, an M_1_R and M_4_R agonist, xanomeline, is now in phase III clinical trials for psychiatric disorders [99].

### 3.6. CHRM5/M_5_R

Patterns of M_5_R expression remain poorly understood due to the paucity of currently available M_5_R-selective agonists and antagonists. M_5_R has been detected in the myenteric plexus of the enteric nervous system (ENS), in midbrain dopaminergic neurons, where it is the predominant muscarinic receptor, and in the cerebral vasculature [61,65,66,98]. In the midbrain, M_5_R activation is essential for neuronal excitability in the substantia nigra pars compacta, and its activation in the striatum modulates dopamine release, suggesting a role for M_5_R in maintaining a balance between the central cholinergic and dopaminergic systems [66]. M_5_R mRNA (*CHRM5*) has been detected in many human tissues, most notably the testes, placenta, thyroid gland, and small intestine, although the significance of its expression in those tissues is uncertain [63,65].

Experimental evidence indicates that M_5_R plays a role in enhancing gastric acid secretion via promotion of histamine secretion from enterochromaffin-like (ECL) cells. Compared to control mice, following treatment with the muscarinic receptor agonist carbamylcholine, M_5_R-deficient mice exhibited significantly reduced gastric acid and histamine secretion in vivo [53]. *Chrm5* was detected in whole stomach samples removed from wild-type mice, but not in samples of fundic or antral gastric mucosa, suggesting M_5_R is expressed in the underlying ENS [53]. Based on these findings, it is plausible that enteric neurons directly or indirectly modulate paracrine release of neuropeptides, which can then stimulate ECL cells to release histamine that, in turn, stimulates acid secretion from gastric parietal cells. Given the recent discovery that glial cells in the myenteric plexus of the ENS express M_3_R and M_5_R, further research is needed to determine the precise role of the ENS in the cholinergic regulation of gastric acid secretion [61].

## 4. Differential Role of Muscarinic Receptor Subtype Activation in GI Cancers

### 4.1. Overview

As discussed above, in terms of physiological and pathophysiological GI epithelial cell function, M_1_R and M_3_R expression and activation have been studied to the relative exclusion of the other muscarinic receptor subtypes. To some extent, this was driven by the initial availability of animal models, e.g., *Chrm1* and *Chrm3* knockout mice with deficient expression alone or in combination of M_1_R and M_3_R [80]. Our understanding of the relative roles played by each of the five muscarinic receptor subtypes in GI neoplasia is likely to be extended now that knockout and transgenic mice with varying expression of M_2_R, M_4_R, and M_5_R are also available. Below, we consider the similarities and differences between M_1_R and M_3_R expression and activation in gastric, pancreatic, and colon cancer (Figure 2).

### 4.2. Gastric Adenocarcinoma

ACh and muscarinic receptor subtypes play prominent roles in the initiation and progression of gastric cancer; differential effects depend on the predominant muscarinic receptor subtype activated. Recent work indicates a likely oncogenic role for the ACh-M_3_R interaction; human gastric adenocarcinoma cells overexpress M_3_R, and expression levels correlate with cancer stage and the presence of lymph node metastases [100]. Conversely, M_3_R deficiency inhibits human gastric adenocarcinoma cell proliferation and induces apoptosis in vitro and in vivo [100]. Even in the absence of stimulation by exogenous ACh, M_3_R antagonism inhibits cancer cell proliferation, suggesting constitutive M_3_R activity or M_3_R activation by autocrine production and release of ACh is sufficient to drive gastric neoplasia [100]; like other GI cancers considered in this review, gastric adenocarcinoma cells can produce and release ACh [58,59]. M_3_R activation results in transactivation of EGFR and downstream activation of MAPK/ERK signaling [35]. In addition, release of ACh from a subset of tuft cells also appears to contribute to pro-proliferative signaling, presumably through a similar mechanism. Unlike in the case of M_3_R antagonism, the use of selective antagonists of M_1_R, M_2_R, and M_4_R failed to attenuate the pro-proliferative effects of ACh on gastric cancer cells [100]. Likewise, others found that gastric stem cells, identified by their expression of the marker Lgr5, co-express *CHRM3*, the gene encoding M_3_R, but not *CHRM1*, *CHRM2*, *CHRM4*, or *CHRM5* [58].

### 4.3. Pancreatic Adenocarcinoma

In pancreatic ductal adenocarcinoma (PDAC), prominent differential actions of muscarinic receptor subtypes are reported, i.e., M_1_R activation protects against neoplasia whereas M_3_R activation promotes pancreatic cancer progression. Renz et al. found that cholinergic signaling through M_1_R suppressed both PDAC development and the cancer stem cell compartment (CSC) [51]. By expanding the CSC, murine subdiaphragmatic vagotomy accelerated PDAC development, reversed by treatment with a muscarinic receptor agonist [51]. Treating mice with a non-selective muscarinic receptor agonist, bethanechol, hindered PDAC progression and prolonged animal survival. The tumor suppressive effects were mediated partially via M_1_R and post-muscarinic receptor MAPK/EGFR and P13K/AKT signaling. In contrast, M_3_R expression correlates with worse clinical outcomes. Zhang et al. detected cytoplasmic M_3_R overexpression in all 58 human PDAC specimens they tested [60]. M_3_R intensity correlated positively with higher PDAC grades, lymph node metastasis, and shorter overall survival. Interestingly, PDAC cells with high levels of M_3_R expression were detected at the invasive tumor front and in metastatic lymph nodes and parasympathetic fibers.

### 4.4. Colon Adenocarcinoma

M_3_R activation and post-receptor signaling is instrumental to colon cancer initiation, invasion, and metastasis. M_3_R is overexpressed in colon cancer, a finding that correlates with poor prognostic features including increased tumor burden, invasion, and metastasis [62,101,102]. M_3_R signal transduction, via the EGFR/ERK and PKC/p38 MAPK pathways, results in the induction and release of selected matrix metalloproteinases (MMP1, MMP7, and MMP10), collagenases that facilitate cell invasion by breaking down the extracellular matrix [103,104]. Additional studies showed that ACh-induced MMP1 expression and colon cancer cell invasion can be abolished by pre-treatment with inhibitors of muscarinic receptor or MMP1 activation [12,105].

Colon cancer initiation is associated with altered functioning of immunocytes in the tumor microenvironment; these immunocytes also express muscarinic receptors (Table 1) [1]. Human T cells release ACh for autocrine and paracrine signaling, a process promoted by activation of M_3_R, T cell activation by immunomodulators (e.g., lipopolysaccharide), and activation of adhesion molecules on the T cell surface; production and secretion of ACh was also identified in human B cells, macrophages, and dendritic cells [1,106]. Given the immune cell-rich environment of the GI tract, particularly the colon, it is plausible that regulation of the immune system via M_3_R also modulates carcinogenesis, for example by producing cytokines that facilitate perineural invasion or otherwise enhancing the tumor microenvironment [107].

A detailed mechanistic understanding of how luminal bile acids, long associated with colon cancer risk, promote colon neoplasia remains elusive. Several lines of investigation identify a prominent role for the functional interaction of selected bile acids with muscarinic receptors, primarily M_3_R expressed by normal colon epithelial cells and overexpressed by colon cancer cells [37,40,44]. Recent work suggests that, via post-M_3_R and Wnt/β-catenin signaling, bile acids may induce the transformation of normal human colonic epithelial cells into colon cancer stem cells [108]. Amongst other evidence, this conclusion is supported by bile acid exposure-induced expression of stem cell and epithelial-mesenchymal transition markers [108]. Suppressing M_3_R expression using RNA interference attenuated bile acid-induced expression of markers of colon cancer stem cells. It appears plausible that M_3_R activation by exposure to luminal bile acids may explain, at least in part, the increased incidence of colon cancer in individuals who consume diets high in saturated fats that are known to increase levels of secondary bile acids in the stool [42,103,109,110].

Although M_3_R deficiency attenuates carcinogen-induced colon neoplasia in mice, M_1_R-deficient mice and those deficient in both M_1_R and M_3_R failed to exhibit decreased tumor formation and proliferation [80]. These findings suggest that M_1_R expression/activation suppresses colon cancer formation and may suppress the pro-neoplastic effects of M_3_R expression/activation. Notably, this may open the door to a therapeutic strategy that blocks M_3_R expression/activation while enhancing M_1_R expression/activation to prevent or treat colon neoplasia. In this respect, initial findings in an orthotopic xenograft mouse model suggest that darifenacin, a selective M_3_R antagonist approved in the United States, Canada, and the European Union to treat urinary incontinence, could be repurposed to attenuate the growth of M_3_R-expressing colon cancers [62]. In vitro, darifenacin also appeared to suppress ACh-stimulated colon cancer cell invasion, with a corresponding suppression of *MMP1* mRNA expression, presumably via inhibition of p38, ERK1/2, and Akt signaling [62]. Whether this therapeutic approach prevents or attenuates metastatic disease in mice remains to be determined; current studies were underpowered to answer these questions [62]. Nonetheless, the results of these exploratory preclinical studies appear promising and suggest that clinical trials of FDA-approved M_3_R-selective antagonists are warranted. Although the development of highly subtype-selective muscarinic antagonists is difficult, in part due to the highly conserved amino acid sequence at the ligand-binding site, the recent discovery of several structural features unique to the M_3_R subtype may facilitate drug development [111].

### 4.5. Role of Muscarinic Receptor Activation within the Enteric Nervous System in GI Neoplasia

The enteric nervous system (ENS), the GI tract’s intrinsic nervous system, consists of neurons and glial cells organized into two concentric plexuses around the lumen. ENS neurons and glia participate in muscarinic signaling, and the ENS’s location in the GI milieu uniquely positions it to influence GI cancers [1,112,113]. For example, nerves underlying the gastric mucosa provide much of that region’s ACh, which activates M_3_R to trigger the release of additional neurotrophic factors [112]. In the context of gastric adenocarcinoma, this results in tropism of ENS neurons toward cancer stem cell foci and promotes carcinogenesis, in part by activating downstream pro-proliferative effectors (e.g., Yes-Associated Protein) and optimizing paracrine nerve–cancer interactions in the tumor microenvironment [59,112,113].

In recent years, attention focused on potential roles for the ENS and its muscarinic components in colon carcinogenesis. In addition to stimulating cancer stem cell growth, promoting colon cancer invasion, and even serving as routes for the physical migration and dissemination of colon cancer cells, the ENS promotes colon carcinogenesis via muscarinic interactions with cells in the tumor microenvironment [114,115].

## 5. Conclusions and Future Directions

An enhanced understanding of the biology of muscarinic receptor subtypes and the role their expression and activation plays in GI neoplasia is likely to identify several novel therapeutic approaches. Both orthosteric agents, which compete for binding at the active ligand binding site, and allosteric agents, which bind elsewhere on muscarinic receptors and alter the conformation of the ligand binding site, can be used to modulate muscarinic receptor activity. Existing FDA-approved, orthosteric muscarinic receptor agonists (e.g., bethanechol) or antagonists (e.g., darifenacin) can be repurposed for testing in therapeutic trials for gastric, pancreatic, and colon cancer. However, due to the nearly identical amino acid sequence at the ligand-binding site, the hurdles to developing agents with true muscarinic receptor subtype selectivity may prove insurmountable. Instead, developing agents targeting less conserved allosteric binding sites is likely to offer a more promising path to developing effective therapeutics [93].

Areas for further exploration include a more granular exploration of cell-type specific muscarinic receptor subtype expression (e.g., employing single cell RNAseq) [116], gaining insights into how muscarinic receptor subtypes currently thought to signal by the same pathways have different, sometimes opposing, effects (e.g., M_1_R and M_3_R) [80], and understanding the mechanisms that underly promiscuous coupling of muscarinic receptor subtypes to different G proteins that may alter their downstream signaling and actions [34]. The biological basis for such alterations is unknown and worthy of exploration since the results may apply to GPCRs in general. In this context, it will be important to overcome key experimental limitations, like the difficulty obtaining GPCR-specific antibodies for immunoblotting [117]. Although mRNA expression provides useful information, for many reasons, including post-transcriptional regulation of gene expression, it may not correlate precisely with protein expression, the ultimate functional determinant. Filling these gaps in knowledge and overcoming these technical limitations will allow us to harness the therapeutic potential of targeting muscarinic receptors subtypes to treat GI cancer. From a clinical perspective, advances in this area of investigation are most likely to be impactful by offering novel treatment modalities targeting muscarinic receptor subtypes for cancers resistant to other forms of therapy.

## Figures and Tables

**Table 1:** Neuronal and non-neuronal cells reported to produce and release acetylcholine.

Sources of Acetylcholine		Refs.
**Neurons**		
Autonomic nervous system	Preganglionic sympathetic/parasympathetic neurons	[23]
Peripheral nervous system	Terminal ends of axons at neuromuscular junctions	[23]
Central nervous system	Primarily interneurons	[23,24]
**Non-Neuronal Cells**		
Immunocytes	CD4 + T cells; B cells; NK cells	[25,26,27]
Placental trophoblast		[28]
Keratinocytes		[29]
Cardiomyocytes		[30]
Airway epithelial cells		[31]
Vascular endothelial cells		[32]
Urothelial cells		[33,34]
Cancer cells	Colon, stomach, lung, and others	[12,35,36]

**Table 2:** G-protein coupling, physiological ligands, tissue distribution, and effects of muscarinic acetylcholine receptor subtype activation in health and disease.

MR Subtype (*Gene*)	G-Protein	Ligands	Tissue Distribution	Physiological Actions	Actions in GI Cancers	Refs.
M_1_R (*CHRM1*)	G_q/11_	ACh, BA, cholesterol (allosteric)	Brain, gastric mucosa, respiratory epithelium, skin, melanocytes, immunocytes	Mediates gastric pepsinogen secretion	Protects against PDAC and colon neoplasia	[34,40,41,50,51,52,53]
M_2_R (*CHRM2*)	G_i/o_	ACh	Brain, heart, ENS, gastric mucosa, skin, bladder, melanocytes, smooth muscle, immunocytes	Modulates cardiac rhythm, GI motility	None reported	[34,50,53,54,55,56]
M_3_R (*CHRM3*)	G_q/11_	ACh, BA	Gastric chief and parietal cells, colon epithelial cells, smooth muscle, ENS, brain, skin, melanocytes, immunocytes	Mediates gastric acid and pepsinogen secretion; GI motility	Promotes gastric and colon cancer cell proliferation and PDAC severity	[37,38,50,52,57,58,59,60,61,62]
M_4_R (*CHRM4*)	G_i/o_	ACh	Brain, gastric mucosa, small intestine, skin, melanocytes, immunocytes	Enhances gastric acid secretion; regulates striatal dopamine release	None reported	[34,50,57,63,64]
M_5_R (*CHRM5*)	G_q/11_	ACh	Brain, cerebral vasculature, ENS; mRNA expressed in testes, placenta, thyroid, small intestine, immunocytes	Enhances gastric acid secretion; regulates striatal dopamine release; mediates SNc excitability	None reported	[34,50,53,61,63,65,66]

Refs, references; MR, muscarinic receptor; ACh, acetylcholine; BA, bile acids; ENS, enteric nervous system; GI, gastrointestinal; PDAC, pancreatic ductal adenocarcinoma; SNc, substantia nigra pars compacta.

## Data Availability

No new data were created or analyzed in this study. Data sharing is not applicable to this article.
